# 114 Burn Specific VTE Prophylaxis: Higher Enoxaparin Dose and Anti-Xa Adjustments Is Safe and Potentially Beneficial

**DOI:** 10.1093/jbcr/irae036.113

**Published:** 2024-04-17

**Authors:** Desiree Pinto, Sophia Lee, Cory Johnson, Rola A Halabi, Tuan D Le, Shawn Tejiram, Taryn E Travis, Lauren T Moffatt, Jeffrey W Shupp

**Affiliations:** MedStar Washington Hospital Center, Washington, DC; MedStar Washington Hospital Center, Grand Prairie, Texas; Georgetown University School of Medicine, Washington, DC; MedStar Washington Hospital Center, Georgetown University School of Medicine, Washington, DC; Firefighters’ Burn and Surgical Research Laboratory, Washington, DC; MedStar Health Georgetown University School of Medicine, Washington, DC; MedStar Washington Hospital Center, Washington, DC; MedStar Washington Hospital Center, Grand Prairie, Texas; Georgetown University School of Medicine, Washington, DC; MedStar Washington Hospital Center, Georgetown University School of Medicine, Washington, DC; Firefighters’ Burn and Surgical Research Laboratory, Washington, DC; MedStar Health Georgetown University School of Medicine, Washington, DC; MedStar Washington Hospital Center, Washington, DC; MedStar Washington Hospital Center, Grand Prairie, Texas; Georgetown University School of Medicine, Washington, DC; MedStar Washington Hospital Center, Georgetown University School of Medicine, Washington, DC; Firefighters’ Burn and Surgical Research Laboratory, Washington, DC; MedStar Health Georgetown University School of Medicine, Washington, DC; MedStar Washington Hospital Center, Washington, DC; MedStar Washington Hospital Center, Grand Prairie, Texas; Georgetown University School of Medicine, Washington, DC; MedStar Washington Hospital Center, Georgetown University School of Medicine, Washington, DC; Firefighters’ Burn and Surgical Research Laboratory, Washington, DC; MedStar Health Georgetown University School of Medicine, Washington, DC; MedStar Washington Hospital Center, Washington, DC; MedStar Washington Hospital Center, Grand Prairie, Texas; Georgetown University School of Medicine, Washington, DC; MedStar Washington Hospital Center, Georgetown University School of Medicine, Washington, DC; Firefighters’ Burn and Surgical Research Laboratory, Washington, DC; MedStar Health Georgetown University School of Medicine, Washington, DC; MedStar Washington Hospital Center, Washington, DC; MedStar Washington Hospital Center, Grand Prairie, Texas; Georgetown University School of Medicine, Washington, DC; MedStar Washington Hospital Center, Georgetown University School of Medicine, Washington, DC; Firefighters’ Burn and Surgical Research Laboratory, Washington, DC; MedStar Health Georgetown University School of Medicine, Washington, DC; MedStar Washington Hospital Center, Washington, DC; MedStar Washington Hospital Center, Grand Prairie, Texas; Georgetown University School of Medicine, Washington, DC; MedStar Washington Hospital Center, Georgetown University School of Medicine, Washington, DC; Firefighters’ Burn and Surgical Research Laboratory, Washington, DC; MedStar Health Georgetown University School of Medicine, Washington, DC; MedStar Washington Hospital Center, Washington, DC; MedStar Washington Hospital Center, Grand Prairie, Texas; Georgetown University School of Medicine, Washington, DC; MedStar Washington Hospital Center, Georgetown University School of Medicine, Washington, DC; Firefighters’ Burn and Surgical Research Laboratory, Washington, DC; MedStar Health Georgetown University School of Medicine, Washington, DC; MedStar Washington Hospital Center, Washington, DC; MedStar Washington Hospital Center, Grand Prairie, Texas; Georgetown University School of Medicine, Washington, DC; MedStar Washington Hospital Center, Georgetown University School of Medicine, Washington, DC; Firefighters’ Burn and Surgical Research Laboratory, Washington, DC; MedStar Health Georgetown University School of Medicine, Washington, DC

## Abstract

**Introduction:**

Burn and trauma patients are at high risk of venous thromboembolism (VTE), including deep vein thrombosis (DVT) and pulmonary embolus (PE). VTE prophylaxis guidelines for trauma patients recommends a higher standard dose of enoxaparin and anti-Xa level adjustments in select patients; however, no guidelines specific to burn patients exist. We hypothesize that higher standard doses of enoxaparin and anti-Xa level adjustments will be safe and effective at reducing VTE complications in burn patients.

**Methods:**

This retrospective review included patients admitted to a regional burn center six months prior to and after the initiation of a VTE prophylaxis quality improvement protocol. The protocol doubled the standard dose of enoxaparin, 40 mg daily to 40 mg twice a day, for all burn patients who were > 50 kg and had creatinine clearance > 60mg/dl. Peak anti-Xa level monitoring was indicated if a high-risk factor (TBSA ≥20%, inhalation injury, burn shock, BMI ≥40) was present. Patients on therapeutic anticoagulation or anti-platelet therapy, had a history of chronic kidney disease, decompensated cirrhosis, coagulation disorder, heparin induced thrombocytopenia (HIT), or were admitted for polytrauma were excluded. Efficacy was evaluated by rates of VTE within 31 days of discharge. Safety was evaluated by bleeding complications, including estimated blood loss (EBL), number of transfusions and gastrointestinal (GI) bleeding events. Differences between groups were compared using a two-sample t-test or Wilcoxon rank-sum test. A p-value of < 0.05 signified statistical significance.

**Results:**

The pre-protocol group included 217 patients and the post-protocol group included 207 patients. There were no significant differences in age (45 vs 44 years), BMI (27 vs 26.4 kg/m2), TBSA (5.9 vs 5.3%), IHI (4.6 vs 7%), number of procedures (3 vs 3), mortality (4.1 vs 3%), or ICU length of stay (1.9 vs 1.6 days) between groups. No significant difference in EBL (50 vs 25 cc), number of transfusions (3.3 vs 2.4 units), or GI bleeding events (1 vs 1) was noted between groups, and no patient developed thrombocytopenia or HIT. There were 5 patients (2.3%) with a VTE within 31 days of discharge in the pre-protocol group compared to 1 patient (0.4%) in the post-protocol group (p=0.19). There was not enough power to determine statistically significant VTE prevention.

**Conclusions:**

A lack of VTE prophylaxis recommendations for burn patients leads to variability in protocols among burn centers. These protocols are infrequently studied. This protocol, which doubled the standard dose of enoxaparin and adjusted based on anti-Xa levels for high-risk patients, was safe. Efficacy and applicability to other centers needs further elucidation.

**Applicability of Research to Practice:**

Enoxaparin 40mg twice a day with anti-Xa level adjustments is safe and a promising strategy to reduce the rates of VTE in the burn population without increasing bleeding complications.

